# Emergency Department SpO_2_/FiO_2_ Ratios Correlate with Mechanical Ventilation and Intensive Care Unit Requirements in COVID-19 Patients

**DOI:** 10.5811/westjem.17975

**Published:** 2024-04-18

**Authors:** Gary Zhang, Michael J. Burla, Benjamin B. Caesar, Carolyne R. Falank, Peter Kyros, Victoria C. Zucco, Aneta Strumilowska, Daniel C. Cullinane, Forest R. Sheppard

**Affiliations:** * Maine Medical Center, Department of Surgery, Portland, Maine; † Southern Maine Healthcare, Department of Emergency Medicine, Biddeford, Maine; ‡ University of New England College of Osteopathic Medicine, Biddeford, Maine; § Tufts School of Medicine, Boston, Massachusetts

## Abstract

**Background:**

Patients with coronavirus 2019 (COVID-19) are at high risk for respiratory dysfunction. The pulse oximetry/fraction of inspired oxygen (SpO_2_/FiO_2_) ratio is a non-invasive assessment of respiratory dysfunction substituted for the PaO_2_:FiO_2_ ratio in Sequential Organ Failure Assessment scoring. We hypothesized that emergency department (ED) SpO_2_/FiO_2_ ratios correlate with requirement for mechanical ventilation in COVID-19 patients. Our objective was to identify COVID-19 patients at greatest risk of requiring mechanical ventilation, using SpO_2_/FiO_2_ ratios.

**Methods:**

We performed a retrospective review of patients admitted with COVID-19 at two hospitals. Highest and lowest SpO_2_/FiO_2_ ratios (percent saturation/fraction of inspired O_2_) were calculated on admission. We performed chi-square, univariate, and multiple regression analysis to evaluate the relationship of admission SpO_2_/FiO_2_ ratios with requirement for mechanical ventilation and intensive care unit (ICU) care.

**Results:**

A total of 539 patients (46% female; 84% White), with a mean age 67.6 ± 18.6 years, met inclusion criteria. Patients who required mechanical ventilation during their hospital stay were statistically younger in age (*P* = 0.001), had a higher body mass index (*P* < .001), and there was a higher percentage of patients who were obese (*P* = 0.03) and morbidly obese (*P* < .001). Shortness of breath, cough, and fever were the most common presenting symptoms with a median temperature of 99°F. Average white blood count was higher in patients who required ventilation (*P* = <0.001). A highest obtained ED SpO_2_/FiO_2_ ratio of ≤300 was associated with a requirement for mechanical ventilation. A lowest obtained ED SpO_2_/FiO_2_ ratio of ≤300 was associated with a requirement for intensive care unit care. There was no statistically significant correlation between ED SpO_2_/FiO_2_ ratios >300 and mechanical ventilation or intensive care unit (ICU) requirement.

**Conclusion:**

The ED SpO_2_/FiO_2_ ratios correlated with mechanical ventilation and ICU requirements during hospitalization for COVID-19. These results support ED SpO_2_/FiO_2_ as a possible triage tool and predictor of hospital resource requirements for patients admitted with COVID-19. Further investigation is warranted.

## INTRODUCTION

The coronavirus 2019 (COVID-19) pandemic profoundly impacted hospital systems worldwide. Identifying patients presenting with COVID-19 in the emergency department (ED) at greatest risk for requiring mechanical ventilation or intensive care unit (ICU) care is of paramount importance since this would facilitate more efficient allocation of limited medical resources. Severe COVID-19 infection can be life-threatening and is associated with significant hypoxemia and the development of acute respiratory distress syndrome (ARDS).^1^^,^^2^ Identifying early predictors of respiratory failure and ICU need is vital both for patient care and logistics in the setting of a global pandemic with limited ICU resources.

The pulse oximetry/fraction of inspired oxygen (SpO_2_/FiO_2_ ratio) has previously been used as a predictor of high-flow nasal cannula failure, need for intubation, and mechanical ventilation.^3^ The SpO_2_ value has been demonstrated to be a reliable surrogate for partial pressure of oxygen in the arterial blood (PaO_2_),^4^^,^^5^ and the SpO_2_/FiO_2_ ratio does not require any blood tests. The SpO_2_/FiO_2_ ratio is a non-invasive assessment of respiratory dysfunction that can be quickly obtained at the bedside. Measured at the time of presentation, the SpO_2_/FiO_2_ ratio has been demonstrated to be an independent indication of ARDS development.^6^ The ability to quickly determine required level of care for vulnerable patients is essential to prevent poor outcomes, particularly in resource-limited environments. The COVID-19 pandemic led to ED crowding and a decrease in ventilator and ICU availability.^7^ A validated prognostic indicator tool akin to the systematic inflammatory response syndrome or Sequential Organ Failure Assessment criteria for sepsis^8^ is vital for ED use to identify COVID-19 patients at highest risk of ventilator and ICU need. The SpO_2_/FIO_2_ ratio predictive value has previously been validated in ARDS,^6^ and early measurement may serve as an indicator and triage tool in COVID-19 with regard to respiratory failure/ventilation risk and ICU need.

Our objective in this study was to evaluate ED SpO_2_/FIO_2_ ratios in COVID-19 patients and correlate them with subsequent respiratory failure, necessitating the need for ICU level of care and/or mechanical ventilation during hospitalization. Use of this ratio may help hospital systems more efficiently use resources and effectively prepare for a patient’s need for ICU care or mechanical ventilation.

## MATERIALS AND METHODS

### Study Design and Participant Selection

This was a retrospective study that evaluated admission encounters from both Maine Medical Center (MMC) and Southern Maine Health Care (SMHC). These institutions work closely together, with MMC being the region’s tertiary care center with over 70,000 annual ED visits and a total of 45 multipurpose ICU beds. The SMHC is a community hospital within close proximity to MMC, averaging ≈55,000 total ED visits and nine ICU beds. COVID-19 patients who were ≥18 years old and required admission to either hospital met inclusion criteria. Encounters were collected between March–December 28, 2020; thus, no patients had been vaccinated against COVID-19. Patients were excluded if they did not require admission. This study was performed under approval of the institutions’ review boards.

### Data Variables

We performed retrospective chart review to identify patient demographics, diagnoses, level of hospital care, and hospital outcomes data from electronic health records. The FiO_2_ values were calculated using nasal cannula flow rate.^9^ We recorded the patient’s lowest and highest SpO_2_ and FiO_2_ values in the ED and calculated SpO_2_/FiO_2_ ratios.

### Outcomes

The primary outcome was the need for mechanical ventilation. Secondary outcomes included ICU level of care, ventilator days, in-hospital complications, escalation of care following initial triage, ICU length of stay (LOS), hospital LOS, and in-hospital mortality.

### Analysis

We analyzed data using RStudio 2020 (RStudio Inc, Boston, MA). Descriptive statistics were presented as frequency and percentage. Normally distributed continuous data were reported as mean with SDs, and ordinal non-normally distributed continuous data were described with medians with interquartile ranges. We used multivariable logistic regression to assess the association between either low or high SpO_2_/FiO_2_ ratios within the ED, anticoagulation use, asthma, coronary artery disease (CAD), congestive health failure (CHF), chronic obstructive pulmonary disease (COPD), diabetes, hyperlipidemia, hypertension, and gastroesophageal reflux disease (GERD), or the need for mechanical ventilation, adjusted for age and body mass index (BMI). Bivariable analysis of categorical variables was done using the χ2 test, and nonparametric variables by the Kruskal-Wallis test. Regression models controlled for both age and BMI.

## RESULTS

A total of 539 patients, with a mean age 67.6 ± 18.6 years, met inclusion criteria. Patients were stratified into two cohorts based on the need for mechanical ventilation (Table 1). As shown in the table, patients who required mechanical ventilation during their hospital stay were statistically younger in age (*P* = 0.001), had a higher BMI (*P* < .001), and there was a higher percentage of patients who were obese (*P* = 0.03) and morbidly obese (*P* < .001). Shortness of breath, cough, and fever were the most common presenting symptoms, with a median temperature of 99°F. The average white blood count was higher in patients who required ventilation (*P* = <0.001) (Table 1). Patients requiring mechanical ventilation had higher diagnoses of ARDS (*P* < .001), pneumonia (*P* < .001), shock (*P* < .001), respiratory and renal failure (*P* < .001), and worse hospital outcomes with an in-hospital mortality of 32% vs 8% (*P* < .001) and a median hospital LOS of 17.5 vs 6 days (*P* < .001).

**Table 1.** Baseline characteristics of patients with coronavirus 2019.

Demographic data	Mean ± SD, median, range or n (%)		
	Not mechanically ventilated n = 451	Mechanically ventilated n = 88	*P*-value
Age (median, IQR)	72, 26	66, 19.75	0.001
BMI (median, IQR)	28.9, 9.4	32.3, 10.9	<.001
Gender			
Female	217 (48%)	31 (35%)	0.03
Male	234 (52%)	57 (65%)	0.03
Race			
Asian	11 (2%)	5 (6%)	0.03
Black	31 (7%)	5 (6%)	0.73
Native Hawaiian or other Pacific Islander	1 (0.2%)	0 (0%)	0
Unknown/not reported	2 (0.4%)	2 (2%)	0.003
More than one race	3 (0.6%)	0 (0%)	0.47
White	397 (88%)	73 (83%)	0.20
Other	6 (1%)	3 (3%)	0.13
Ethnicity			
Hispanic or Latino	9 (2%)	3 (3%)	0.56
Not Hispanic or Latino	440 (98%)	84 (95%)	0.10
Unknown/not reported	2 (0.4%)	1 (1%)	0.46
Origin			
Home	282 (63%)	54 (61%)	0.72
Nursing home	61 (14%)	9 (10%)	0.31
Skilled nursing home	31 (7%)	0 (0%)	0.01
Rehab	1 (0.2%)	2 (2%)	0.03
Other^*^	76 (17%)	23 (26%)	0.05
Comorbid conditions			
Alcohol use	23 (5%)	8 (9%)	0.14
Anticoagulation therapy	52 (12%)	13 (15%)	0.44
Asthma	66 (15%)	14 (16%)	0.81
Cerebrovascular accident	41 (9%)	4 (5%)	0.22
COPD	71 (16%)	16 (18%)	0.64
Chronic heart failure	67 (15%)	13 (15%)	1
Chronic kidney disease	73 (16%)	12 (14%)	0.64
Cancer	57 (13%)	9 (10%)	0.44
Coronary heart disease/heart failure	105 (23%)	19 (22%)	0.84
Current smoker	30 (7%)	2 (2%)	0.08
Dementia	75 (17%)	5 (6%)	0.01
Diabetes mellitus	156 (35%)	38 (43%)	0.15
GERD	132 (29%)	26 (30%)	0.85
Myocardial infraction	39 (9%)	5 (6%)	0.36
Hypertension	282 (63%)	57 (65%)	0.72
Hyperlipidemia	222 (49%)	49 (56%)	0.23
Morbidly obese	14 (3%)	11 (13%)	<.001
Obese	81 (18%)	25 (28%)	0.03
Presenting symptoms			
Fever	176 (39%)	34 (39%)	1
Myalgia	72 (16%)	15 (17%)	0.82
Arthralgias	21 (5%)	2 (2%)	0.22
Headache	50 (11%)	4 (5%)	0.09
GI symptoms	140 (31%)	17 (19%)	0.02
Cough	229 (51%)	54 (61%)	0.09
Shortness of breath	253 (56%)	57 (65%)	0.12
Other	233 (52%)	43 (49%)	0.61
Average temperature in the ED ± SD (Fahrenheit)	97.1 ± 12.4, 99.1, 7.3–104.5	98.1 ± 10, 99, 37–103	0.48
WBC count in the ED (median, IQR)	6.2, 4.7	8, 7.4	<.001
Diagnoses			
ARDS	24 (5%)	57 (65%)	<.001
Pneumonia	183 (41%)	60 (68%)	<.001
Neurological diagnoses	128 (28%)	40 (45%)	0.002
Renal diagnoses	129 (29%)	55 (63%)	<.001
Liver diagnoses	44 (10%)	23 (26%)	<.001
Heart diagnoses	168 (37%)	56 (64%)	<.001
Pulmonary diagnoses	280 (62%)	69 (78%)	0.004
Shock	10 (2%)	46 (52%)	<.001
Respiratory failure	153 (34%)	75 (85%)	<.001
Renal failure	29 (6%)	22 (25%)	<.001
ICU			
Patients who required ICU care at any point	75 (17%)	84 (95%)	<.001
Required more than one ICU admissions	2 (0.4%)	6 (7%)	<.001
ICU LOS (median, IQR)	2, 3	13, 16	<.001
Intubated			
Patients who were intubated	0 (0%)	84 (95%)	<.001
Days intubated	n/a	2, 4	
Non-procedure based intubation	0 (0%)	51 (58%)	<.001
Mechanical ventilators			
Ventilator days (median, IQR)	n/a	9, 13	
Required reintubation	n/a	7 (8%)	
Escalation of care from initial triage	60 (13%)	56 (64%)	<.001
Hospital LOS (median, IQR)	6, 6	17.5, 19	<.001
Discharge disposition			
Home or self-care	193 (43%)	8 (9%)	<.001
Home with services	97 (22%)	17 (19%)	0.53
Hospice/palliative care unit	11 (2%)	1 (1%)	0.52
Mental health/psychiatric hospital	8 (2%)	0 (0%)	0.18
Nursing home	17 (4%)	1 (1%)	0.16
Other	62 (14%)	34 (39%)	<.001
Rehab	15 (3%)	24 (27%)	<.001
Skilled nursing facility	48 (11%)	3 (3%)	0.02
In-hospital mortality	36 (8%)	28 (32%)	<.001

^*^ Other includes homeless, transfers in, group home, Primary care physician follow up, mental health facility.

*BMI*, body mass index; *IQR*, interquartile range; *COPD*, chronic obstructive pulmonary disease; *GERD*, gastroesophageal reflux disease; *WBC*, white blood count; *ED*, emergency department; *ARDS*, acute respiratory disease syndrome; *GI*, gastrointestinal; *ICU*, intensive care unit; *LOS*, length of stay.

The SpO_2_/FiO_2_ ratios in the ED and their associations with mechanical ventilation or need for ICU care are presented in Table 2. A highest obtained ED SpO_2_/FiO_2_ ratio of 300 or below was statistically associated with a requirement for mechanical ventilation during hospitalization. A lowest obtained ED SpO_2_/FiO_2_ ratio of 300 or below was statistically associated with a requirement for ICU care during hospitalization. There was no statistically significant relationship between ED SpO_2_/FiO_2_ ratios above >300 and mechanical ventilation or ICU level of care.

**Table 2.** SpO_2_/FiO_2_ ratios and their association with intensive care unit or mechanical ventilation needs.

Variable SpO_2_/FiO_2_ ratios^*^	No mechanical ventilation N (%)	Required mechanical ventilation N (%)	95% CI	OR	*P*-value
Lowest ED SpO_2_/FiO_2_					
0–100	18 (4)	13 (18)	2.4–10.9	5.1	<.001
101–200	5 (1)	3 (4)	0.75–12.6	3.1	0.05
201–300	37 (9)	14 (20)	1.2–4.7	2.4	0.005
301–400	113 (27)	19 (27)	0.6–1.8	1.0	0.86
401–500	251 (59)	22 (31)	0.2–0.5	0.31	<.001
Highest ED SpO_2_/FiO_2_					
0–100	7 (2)	7 (10)	2.2–19.2	6.5	<.001
101–200	4 (1)	3 (4)	1.0–21.2	4.6	0.05
201–300	14 (3)	11 (15)	1.7–8.1	3.7	0.002
301–400	106 (25)	20 (28)	0.72–2.2	1.3	0.47
401–500	293 (69)	30 (42)	0.2–0.6	0.3	<.001
Variable SpO_2_/FiO_2_ ratios^*^	No ICU admission N (%)	ICU admission N (%)	95% CI	OR	*P*-value
Lowest ED SpO_2_/FiO_2_					
0–100	8 (2)	23 (17)	3.8–20	8.8	<.001
101–200	1 (0.3)	7 (5)	2.3–158	19.2	<.001
201–300	27 (8)	24 (18)	1.4–4.5	2.5	0.001
301–400	94 (26)	36 (26)	0.6–1.5	0.93	0.66
401–500	228 (64)	47 (34)	0.2–0.5	0.32	<.001
Highest ED SpO_2_/FiO_2_					
0–100	3 (1)	11 (8)	2.8–10	10.3	<.001
101–200	0 (0)	7 (5)	2.3–19	19.2	<.001
201–300	11 (3)	14 (10)	1.3–2.8	2.80	0.01
301–400	88 (25)	38 (28)	0.78–1.2	1.21	0.66
401–500	256 (72)	67 (49)	0.26–0.39	0.39	<.001

^*^ For patients who had ED SpO_2_/FiO_2_ values.

*CI*, confidence interval; *OR*, odds ratio; *ED*, emergency department; *ICU*, intensive care unit.

Chronic obstructive pulmonary disease was a confounding factor for COVID-19 patients who required mechanical ventilation (adjusted R^2^ value = 0.1132; *P* < .001). No statistically significant associations were identified between the following co-morbidities: anticoagulation use; asthma (adjusted R^2^ = 0.096, *P* = 0.75); CAD (adjusted R^2^ = 0.102; *P* = 0.07); CHF (adjusted R^2^ = 0.096; *P* = 0.95); diabetes (adjusted R^2^ = 0.10; *P* = 0.07); hyperlipidemia (adjusted R^2^ = 0.11; *P* = 0.08); hypertension (adjusted R^2^ = 0.096; *P* = 0.58); and GERD (adjusted R^2^ = 010; *P* = 0.28) for the requirement of mechanical ventilation.

## DISCUSSION

This study demonstrated that the highest obtained ED SpO_2_/FiO_2_ ratio of 300 or below correlated with the need for mechanical ventilation during hospitalization. Additionally, a lowest obtained ED SpO_2_/FiO_2_ ratio of 300 or below was associated with a requirement for ICU-level care. Although COPD was a confounding factor for patients requiring mechanical ventilation, other co-morbidities were not independently associated with higher rates of mechanical ventilation and the ED SpO_2_/FiO_2_. This suggests that the SpO_2_/FiO_2_ ratio can be used as a prognostic indicator to stratify severity of illness in patients with COVID-19 during their initial evaluation in the ED. Since the SpO_2_/FiO_2_ ratio is non-invasive and can be quickly obtained and trended during a patient’s evaluation, this ratio could be an important factor in patient triage and disposition.

Multiple prognostic indicators have been proposed in the previous literature to help stratify ARDS severity and predict outcomes.^10^^–^^13^ The PaO_2_:FiO_2_ (P:F) ratio is a widely used measure of ARDS severity; however, multiple studies have shown that the P:F ratio is not an independent predictor of mortality.^10^^–^^13^ Another prognostic tool, the oxygenation index, (OI [FIO_2_/PaO_2_ × mean airway pressure × 100]) has been demonstrated to be an independent risk factor for mortality in adults with ARDS,^11^^,^^12^ but it requires mechanical ventilation and arterial blood gas analysis for calculation. Oxygen saturation index (OSI [FIO_2_ × mean airway pressure × 100)/SaO_2_]) is a measure that correlates to OI and is an independent predictor of clinical outcomes.^12^ Although OSI calculation does not require blood analysis, it still requires mechanical ventilation. Another prognostic tool, the Lung Injury Prediction Score (LIPS), has applicability in the ED.^13^ However, the LIPS tool requires a detailed past medical history (e.g, alcohol use disorder) and the patient’s pH, requiring a blood gas. Although all these tools provide some prognostic value, each has limitations, resulting in barriers to deployment for triaging patients in the ED.

In contrast, the SpO_2_/FiO_2_ ratio requires no blood tests and is quickly and easily obtained at the bedside. Measured at the time of presentation, it has been shown to be an independent indication of ARDS development.^6^ This study suggests that the SpO_2_/FIO_2_ ratio may offer an estimate of disease severity in patients with COVID-19 before progression to overt respiratory failure, serving as a triage tool to identify those at greatest risk for needing mechanical ventilation and critical care. The SpO_2_/FiO_2_ ratio can be used as a tool or part of a protocol to assess whether a patient meets transfer criteria within a hospital system. Many regional health systems operate under a “hub and spoke” model where a large central institution supports a network of smaller hospitals. Rapid identification of patients at risk for decompensation and with need for higher level care would facilitate access to limited critical care resources while also decreasing the incidence of over-triage to the hub hospital.

## LIMITATIONS

The study is retrospective with inherent limitations in controlling confounding variables. The cohort was limited to one hospital system, and thus cannot account for practice variations in other healthcare systems. The hospitals evaluated in this study may have had different criteria for ICU admission. Additionally, FiO_2_ values were based largely on nasal cannula flow rates; limiting to high flow nasal cannula would permit more accurate FiO_2_ but would also limit applicability. At the time of data collection, no patients were vaccinated, thus limiting the applicability of findings to populations with some form of COVID-19 vaccination.

## CONCLUSION

In summary, ED SpO_2_/FiO_2_ ratios correlate with mechanical ventilation and ICU requirements during hospitalization for COVID-19 infection. These results support ED SpO_2_/FiO_2_ as a triage tool and predictor of hospital resource requirements for patients admitted with COVID-19. Further study is required with a prospective analysis assessing accuracy of the SpO_2_/FiO_2_ ratio in predicting mechanical ventilation and need for ICU-level care.
